# Influence of Intestinal Indigenous Microbiota on Intrafamilial Infection by *Helicobacter pylori* in Japan

**DOI:** 10.3389/fimmu.2018.00287

**Published:** 2018-02-21

**Authors:** Takako Osaki, Cynthia Zaman, Hideo Yonezawa, Yingsong Lin, Masumi Okuda, Eriko Nozaki, Fuhito Hojo, Satoshi Kurata, Tomoko Hanawa, Shogo Kikuchi, Shigeru Kamiya

**Affiliations:** ^1^Department of Infectious Diseases, Kyorin University School of Medicine, Tokyo, Japan; ^2^Department of Public Health, Aichi Medical University School of Medicine, Aichi, Japan; ^3^Department of Pediatrics, Aichi Medical University School of Medicine, Aichi, Japan; ^4^Department of General Medicine and Community Health Science, Hyogo College of Medicine, Hyogo, Japan; ^5^Core Laboratory for Proteomics and Genomics, Kyorin University School of Medicine, Tokyo, Japan; ^6^Graduate School of Medicine, Institute of Laboratory Animals, Kyorin University, Tokyo, Japan

**Keywords:** *Helicobacter pylori*, intrafamilial infection, intestinal microbiota, mother-to-child transmission, beta-diversity, Manhattan distance

## Abstract

*Helicobacter pylori* is a causative pathogen of chronic gastritis, gastric ulcer disease, and gastric cancer. Humans are known to be a natural host for *H. pylori* and tend to acquire the pathogen before the age of 5 years. The infection may then persist lifelong if eradication therapy is not applied. One of the modes of transmission of *H. pylori* is between family members, and therefore, the presence of infected family members is an important risk factor in children. However, other environmental factors have not been fully analyzed. The present study was performed to clarify whether and to what extent intestinal microbiota affect *H. pylori* intrafamilial infection. The fecal specimens from *H. pylori-*infected infants and *H. pylori-*infected and non-infected family members were collected in cohort studies conducted by Sasayama City, Hyogo Prefecture from 2010 to 2013. In total, 18 fecal DNA from 5 families were analyzed. Samples were amplified using 16S rRNA universal primers, and the amplicons were sequenced using the Ion PGM system. Principal-coordinate analysis demonstrated that there was no difference in intestinal microbiota between *H. pylori-*positive and *H. pylori-*negative groups. In intrafamilial comparison tests, the Manhattan distance of intestinal microbiota between the *H. pylori-*infected infant proband and *H. pylori-*negative mother was nearest in the family with low intestinal microbial diversity. However, in the family with the highest intestinal microbial diversity, the nearest Manhattan distance was shown between the *H. pylori-*infected infant proband and *H. pylori-*infected mother. The results in this study showed that the composition of the intestinal microbiota was very similar between members of the same family, and as such, colonization with organisms highly similar to the infected parent(s) may be a risk factor for *H. pylori* infection in children.

## Introduction

Humans are the natural host for *Helicobacter pylori* and more than half of the world’s population are infected with this microorganism. Long-term infection with *H. pylori* significantly increases the risk of developing site-specific diseases, such as peptic ulcer disease (1), gastric adenocarcinoma (2, 3), and mucosa-associated lymphoid tissue lymphoma (3, 4).

*Helicobacter pylori* is most commonly acquired and colonize the human stomach up to the age of 5 years (5), and infection may persist lifelong if eradication therapy is not applied. In the developing countries where *H. pylori* is endemic in the environment, fecal–oral infection through drinking of water contaminated by *H. pylori*-infected patients’ feces is an important infection route (6). On the other hand, in the developed countries where the mode of transmission of *H. pylori* is mainly between family members, the presence of infected family members is an important risk factor in children (7–9). Recently, *H. pylori* prevalence in the younger age groups has decreased in Japan (10), and mother-to-child transmission has become the major infection route detected in Japanese children (9, 11–13).

After birth, infants are constantly exposed to person-to-person and environmental contact with microbes, and the development of the indigenous microbiota begins. Family microbiota are shared between parents and infants, which plays an important role in the development of the infant microbiome. Current research suggests that the disorder of microbiota induces the gastrointestinal tract.

In our previous study (8), *H. pylori* infection in children was associated with infection in their mothers or fathers, but not in siblings or grandparents. In addition, we reported on intrafamilial infection with *H. pylori* by mother-to-child or father-to-child transmission in three families using multilocus sequencing type analysis of fecal specimens (8). In the present study, it was thought that the mother- or father-to-child transmission of *H. pylori* was more frequent than sibling-to-sibling transmission in this cohort. We focused on the comparison of microbiota between index children with *H. pylori* infection and siblings without *H. pylori* infection. Moreover, we also investigated the diversity of the microbiota between the index child and infected mother or father and whether this affected the routes of *H. pylori* infection for index children.

## Materials and Methods

### Subjects

Our previous Sasayama study from 2010 to 2013 in Sasayama city, Hyogo, Japan was undertaken and detailed in previous reports (8, 10, 14). For the diagnosis of *H. pylori* infection, a TestMate Pylori Antigen enzyme immunoassay T (Wakamoto Co., Ltd., Kanagawa, Japan) and real-time polymerase chain reaction detection of *H. pylori* DNA by the 16S rRNA gene of *H. pylori*-targeted primers were used (15, 16).

In this study, we analyzed a total of 18 fecal specimens that were collected from 5 *H. pylori*-infected children and their family members.

A modified protocol of the one used in the Sasayama study was undertaken in accordance with the Declaration of Helsinki with approval from the Ethics Committees of Kyorin University, Tokyo.

### Extraction of Fecal DNA

Fecal specimens were stored at −80°C until use. The DNA from the fecal samples was recovered using the QIAamp DNA Stool kit (Qiagen, Germantown, MD, USA) according to the manufacturer’s instructions with some modification (10). Two hundred milligrams of the fecal specimens was weighed in a 2 ml microcentrifuge tube containing 0.3 g glass beads, and 180 µl of ATL lysis buffer solution was added (Qiagen). The suspension was mixed using a vortex mixer followed by bead beating three times for 30 s at a setting of 4,200 rpm using a bead beater (Yasui Kikai, Tokyo, Japan). In addition, 20 µl of proteinase K was added to the mixture. After heating at 56°C for 30 min, the bacterial cells in the samples were treated by bead beating in the same manner. After destruction of bacterial cells, total DNA was collected using a Qiagen column and purified according to the manufacturer’s instructions. The DNA was extracted in 200 µl of AE buffer (Qiagen) with 400 µl of ethanol and 20 µl of 3 M sodium acetate and kept at −20°C for 14–16 h for ethanol precipitation. The precipitated DNA was collected by centrifugation for 20 min at 20,000 × *g*.

### 16S Metagenomic Analysis

Each DNA specimen was amplified using the Ion 16S™ Metagenomics Kit (Thermo Fisher Scientific, Bremen, Germany). The amplicons were purified and prepared for the sequencing library by using the Ion Plus Fragment Library Kit (Thermo Fisher Scientific) according to the manufacturer’s instructions. The library was sequenced by using the Ion PGM system (Thermo Fisher Scientific) and the Ion PGM Hi-Q sequencing kit following the protocol of the kit (17, 18).

The operational taxonomic unit of each sequence was determined by the Ion torrent server with the Greengenes database (19). The minimum alignment value calculated for each aligned read in the analysis was 90.0% for the coverage between hit and query. The number of unique reads needed for the read to be valid was 10. To make a genus ID, a percentage identity value of 97.0% was used, and to identify a species ID, 99.0% was used. If more than one species or genus were found within 0.2% difference from each other, they were each reported as a “slash ID”. The server system was equipped with QIIME (20) for the analysis of microbiota and Emperor (21) for the visualized beta diversities among sequenced samples.

### Statistical Analysis

Statistical analysis was performed using the software KaleidaGraph (Hulinks Inc., Tokyo, Japan). Mean values were compared among subjects using the Kruskal–Wallis test between all groups. For comparing categorical data, the χ^2^ test was performed. A probability value (*p* value) less than 0.05 was considered statistically significant.

## Results

### *Helicobacter pylori* Infection of Family Members

The 18 fecal specimens from 5 *H. pylori*-infected children and their family members were tested for microbiota analysis. The details are shown in Table 1. In total, five families, containing four *H. pylori*-infected mothers and three *H. pylori*-infected fathers, four *H. pylori*-uninfected siblings, and one each uninfected father and mother, as well as five index children were tested. All five *H. pylori*-infected index children had a *H. pylori*-infected mother and/or father but no *H. pylori*-infected sibling.

**Table 1:** *Helicobacter pylori* infection-positive children and their families.

	*H. pylori* infection positive	*H. pylori* infection negative
Family 1	Index child and father	Mother and sibling
Family 2	Index child and mother	Sibling
Family 3	Index child and mother	Father and sibling
Family 4	Index child, father, and mother	
Family 5	Index child, father, and mother	Sibling

### Relative Abundance of Phyla in Intestinal Microbiota of Subjects Depending on *H. pylori* Status and Age

**Figure 1:**
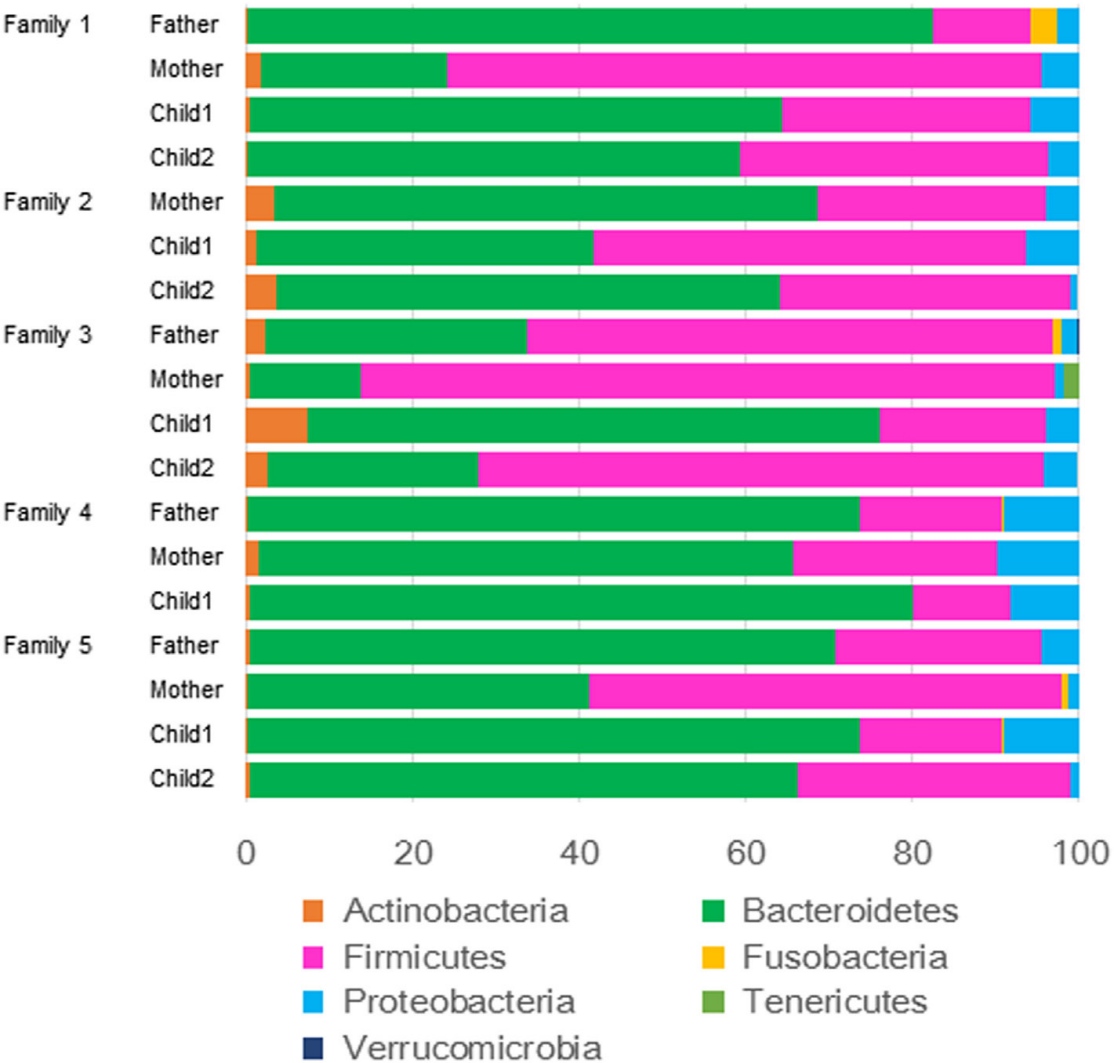
Relative abundance at the phylum level (97% similarity) of intestinal microbiota of each family member.

**Figure 2:**
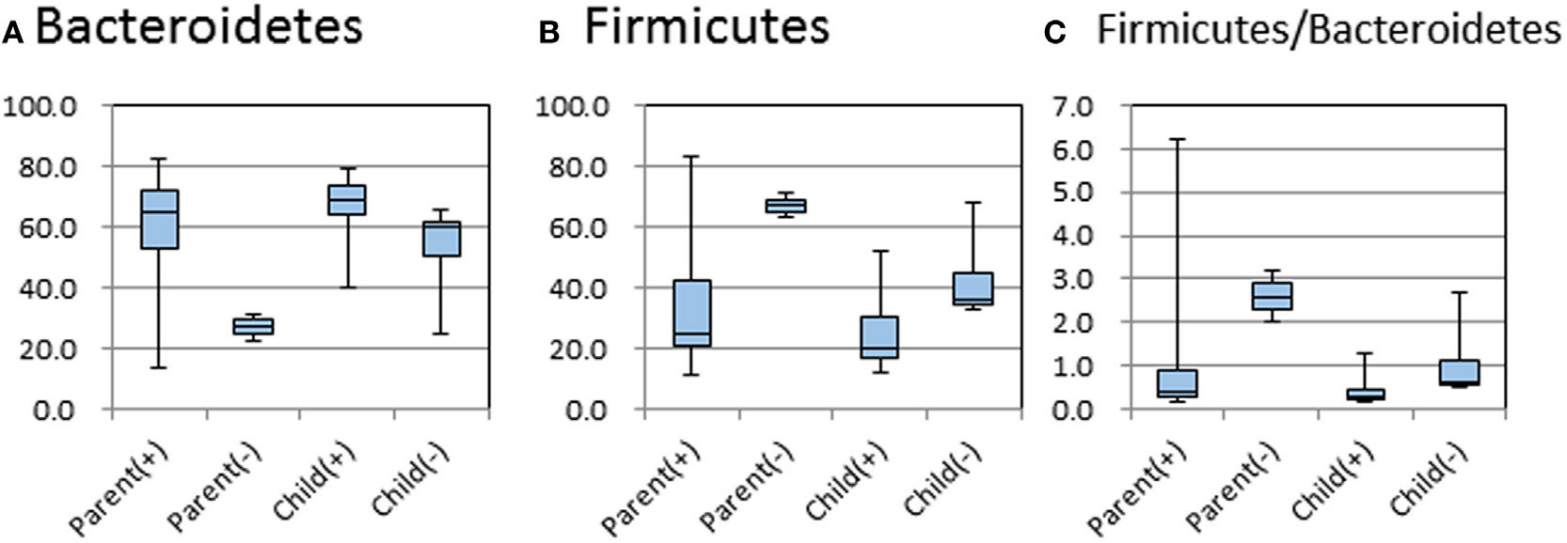
Relative abundances of Bacteroidetes and Firmicutes, and the relative ratio of Firmicutes/Bacteroidetes in microbiota of parents and children in five families with or without *Helicobacter pylori* infection. (+) and (−) indicate *H. pylori* infection positive and negative, respectively. The middle line in the box plot represents the median value, and the box is drawn from 25 to 75% quartiles. Whiskers show minimum and maximum values, and the ends of the whiskers represent the non-outlier range.

The relative abundance of phyla in the feces of each family member is shown in Figure 1. The dominant phyla of the fecal microbiota were Bacteroidetes (12/18 cases) and Firmicutes (6/18 cases). For the comparative study of intestinal microbiota concerning *H. pylori* infection and age, the subjects were divided into *H. pylori* infected and non-infected, and the subgroups were also divided into parent and child groups. In these four groups of subjects, the abundance of Bacteroidetes or Firmicutes and the ratios of Firmicutes/Bacteroidetes were compared (Figure 2). There were no significant differences in the abundance of both phyla by the Kruskal–Wallis test.

### Differences in Relative Abundances of Bacterial Families, Genera, and Species in the Intestinal Microbiota of Subjects

**Figure 3:**
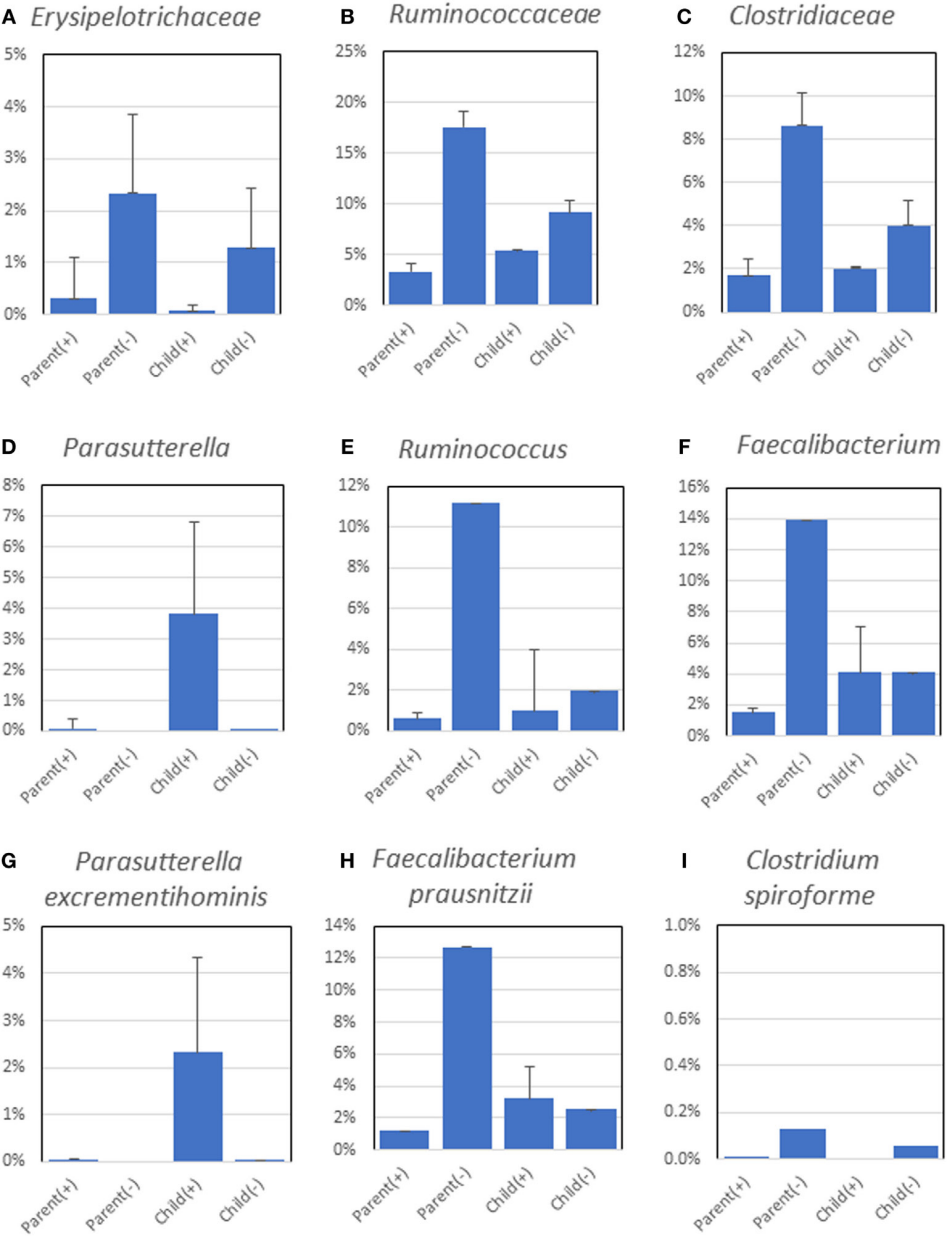
Significant differences (*p* values < 0.05) in relative abundances of *Erysipelotrichaceae* **(A)**, *Ruminococcaceae* **(B)**, *Clostridiaceae* **(C)**, *Parasutterella* **(D)**, *Ruminococcus* **(E)**, *Faecalibacterium* **(F)**, *Parasutterella excrementihominis* **(G)**, *Faecalibacterium prausnitzii* **(H)**, and *Clostridium spiroforme* **(I)** composing fecal microbiota. The abundances of the bacteria in bacterial family **(A–C)**, genus **(D–F)**, and species **(G–I)** were used to determine the statistical significance of differences between groups by the Kruskal–Wallis test.

The average relative abundance of each bacterial family, genus, and species was compared among the four groups. At the family level, abundances of *Erysipelotrichaceae, Clostridiaceae*, and *Ruminococcaceae* were higher in the *H. pylori*-negative groups than those in the *H. pylori* positive groups (Figures 3A–C). At the genus level, a significantly higher abundance of *Parasutterella* was detected in *H. pylori*-positive children (Figure 3D). Higher abundances of *Ruminococcus* and *Faecalibacterium* were also detected in *H. pylori*-negative parents (Figures 3E,F). At the species level, significantly higher abundance of *Parasutterella excrementihominis* was detected in *H. pylori*-positive children (Figure 3G). Higher abundances of *Faecalibacterium prausnitzii* were detected in the microbiota of *H. pylori*-negative parents (Figure 3H), and *Clostridium spiroforme* was seen in abundance in both *H. pylori*-negative parents and children (Figure 3I). *Turicibacter sanguinis* was detected only in the microbiota of *H. pylori*-negative parents (two cases). *H. pylori* was not detected in *H. pylori*-negative and -positive subjects at all from fecal microbiota.

**Figure 4:**
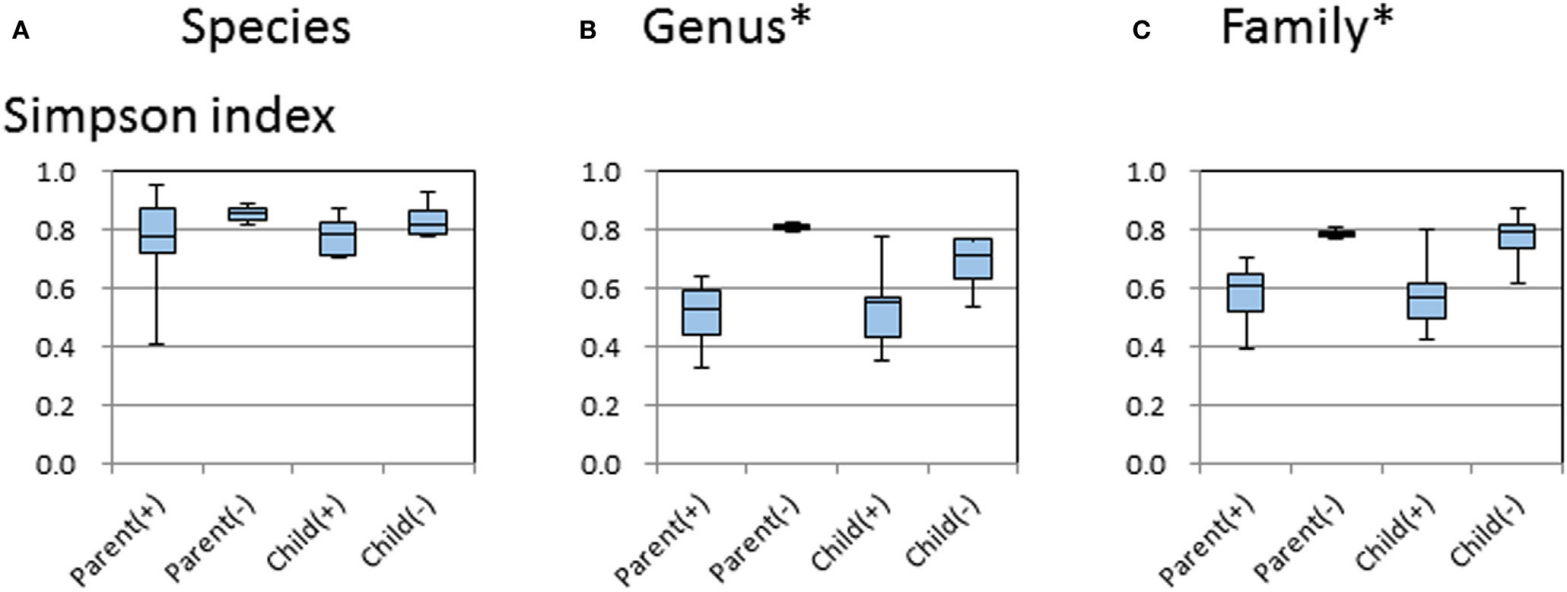
Alpha diversity plots of Simpson index measures at species level **(A)**, genus level **(B)**, and family level **(C)** for the 18 subjects grouped by positive (+) or negative (−) *Helicobacter pylori* status and by the parent or child in the family. The middle line in the box plot represents the median value, and the box is drawn from 25 to 75% quartiles. Whiskers show minimum and maximum values, and the ends of the whiskers represent the non-outlier range. *p*-Values of 0.05 (*) by the Kruskal–Wallis test were used to determine the statistical significance of differences between groups.

The diversity indices were calculated for the entire sample set according to the *H. pylori* status and age groups. As shown in Figure 4, Simpson’s indices were significantly higher in samples from *H. pylori*-negative parents than in those from *H. pylori*-positive parents. These results remained significant even when the comparisons were performed at family and genus levels (Figure 4).

**Figure 5:**
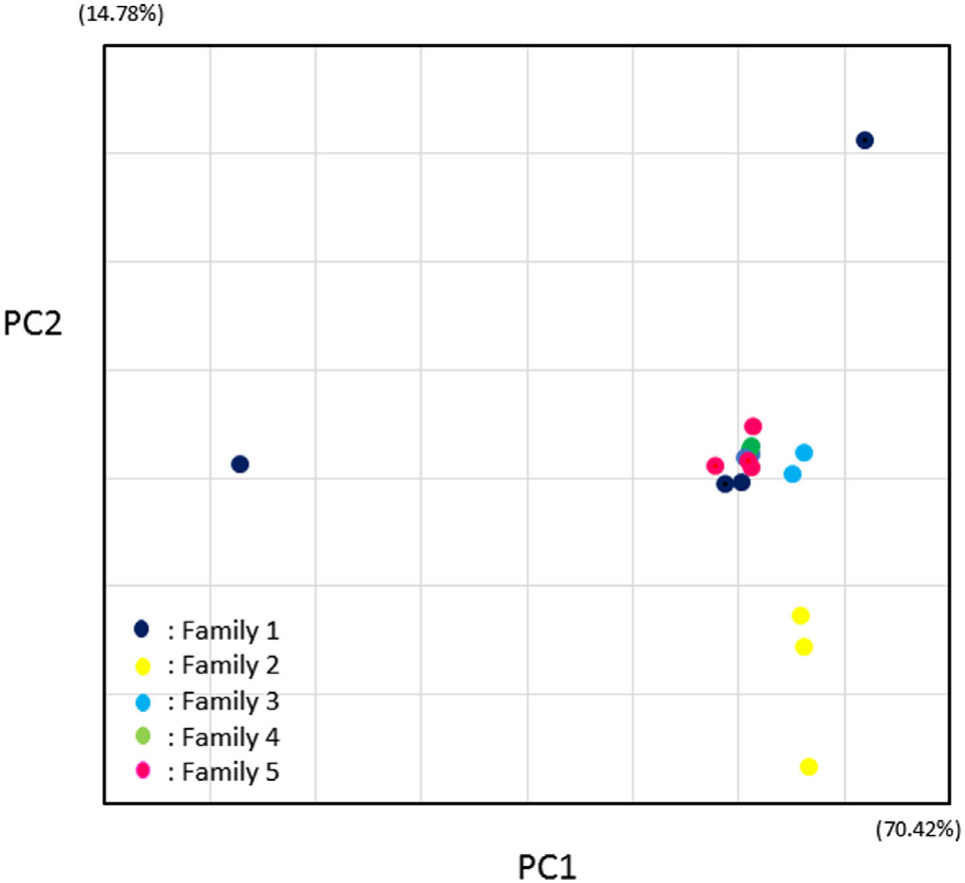
Principal-coordinate analysis of Manhattan distances highlighting differences in intestinal microbiota of family members at the species level. PC1 and PC2 represent the first two highest discriminating axes. The percentage variation explained by each PC axis is indicated. Differently colored symbols represent each family.

Principal-coordinate analysis (PCoA) of Manhattan distances highlighting similarities between fecal specimens of the 16 family members is shown in Figure 5 and Table 2.

**Table 2:** Similarity of microbiota between index child and each family member using Manhattan distance.

Family	*H. pylori* infection		Manhattan distance of each family member at bacterial family level			
**Family 1**			**Index child**	**Father**	**Mother**	**Sibling**
	Index child	+	0			
	Father	+	$\underline{\text{308758}}$	0		
	Mother	(−)	$\underline{\text{127072}}$	288172	0	
	Sibling	(−)	1207218	$\underline{\underline{\text{1496290}}}$	1303190	0

**Family 2**			**Index child**	**Mother**	**Sibling**	
	Index child	+	0			
	Mother	+	$\underline{\text{213363}}$	0		
	Sibling	(−)	204327	$\underline{\underline{\text{107814}}}$	0	

**Family 3**			**Index child**	**Father**	**Mother**	**Sibling**
	Index child	+	0			
	Father	(−)	178915	0		
	Mother	+	$\underline{\text{69034}}$	201923	0	
	Sibling	(−)	291835	306308	$\underline{\underline{\text{322567}}}$	0

**Family 4**			**Index child**	**Father**	**Mother**	
	Index child	+	0			
	Father	+	51403	0		
	Mother	+	$\underline{\text{24078}}$	60805	0	

**Family 5**			**Index child**	**Father**	**Mother**	**Sibling**
	Index child	+	0			
	Father	+	$\underline{\text{36008}}$	0		
	Mother	+	92170	93518	0	
	Sibling	(−)	84593	$\underline{\underline{\text{76961}}}$	49063	0

PC1 and PC2 represent the first two highest discriminating axes. At both levels of species and family, family 2 was divided and separated from other families (Figure 5). PCoA plots of other families showed with very near Manhattan distances with exceptional family 1 members, and the beta diversities of the fecal microbiota in families 3, 4, and 5 were close to each other. These results showed that fecal microbiota were similar not only between members of the same family but also between other families.

Manhattan distances at the bacterial family level between family members in each family are shown in Table 2. The Manhattan distances between the index child and other family members show the similarity between the microbiota. Four mothers (underlined in Table 2) and one father (underlined in Table 2) show the highest similarities to the *Helicobacter* positive index children in the five families. However, one (family 1) of four mothers was *H. pylori* negative, and the other four members were *H. pylori* positive.

In comparing distances from *H. pylori*-positive parent to children in family 1, the distance between the *H. pylori*-positive parent to the index child (underlined in Table 2) is shorter than that between mother and sibling (double underlined in Table 2).

## Discussion

Many factors affect the composition of intestinal microbiota (22), and the influence of diet, lifestyle, age, gender, and geography is well known. In this study, it was thought that the background of all subjects was close, since they all lived in the same location (Sasayama city, Japan) and had a similar lifestyle.

The normal human intestinal microbiota comprises two major phyla, the Gram-negative Bacteroidetes and the Gram-positive Firmicutes. In this study, Bacteroidetes existed dominantly in 12 subjects and Firmicutes existed dominantly in other 6 subjects. There were no significant differences in the abundance of dominant phyla between *H. pylori*-positive and -negative groups in children. It is well known that the ratio of Firmicutes/Bacteroidetes is related to obesity and the body mass index (BMI) of human and animal subjects and that the eradication of *H. pylori* infection induces an increase in BMI (23, 24). There have been no previous reports into the ratio of Firmicutes/Bacteroidetes in *H. pylori*-infected patients. In this study, we compared the ratio between the four groups comprising infected and non-infected children and parents. There was no significant difference in the abovementioned ratio between the four groups. However, in the parents, we only had two *H. pylori* infection-negative subjects. Therefore, the higher ratio of Firmicutes/Bacteroidetes in *H. pylori*-negative parents compared to *H. pylori*-positive parents shown was not significant due to sample size. As a limitation in our study, we were limited by the small sample size, and the number of subjects was very small especially in some subgroups. Additional study using much more samples need to be done in the future.

The average relative abundance numbers for each bacterial family, genus, and species was compared among the four groups. A higher abundance of *F. prausnitzii* was detected in the microbiota of *H. pylori*-negative parents. *F. prausnitzii* has been reported to be one of the bacterial species possessing anti-inflammatory properties (25, 26), and higher abundances of this species were detected preferentially in Japanese subjects with lean body mass (27).

*Parasutterella excrementihominis*, a member of family *Alcaligenaceae*, was only detected from the microbiota of *H. pylori*-positive children. *P. excrementihominis* was first isolated from the feces of a healthy Japanese male by Nagai et al (28), and they reported that this new species showed more than 98% 16S rRNA gene sequence similarity to some of the human intestinal uncultured clones reported by several groups in the US and other countries. This indicates that these bacteria are likely to be common members of the human intestinal microbiota. However, we cannot shed more light on why it was detected only in *H. pylori*-positive children in this study, and further studies are needed to clarify the differences in microbiota between *H. pylori*-positive and -negative children using a larger subject population.

The average Simpson index values of the four groups were also compared by the Kruskal–Wallis test. Higher average values of Simpson index in the *H. pylori*-negative groups were detected by comparison at the bacterial genus and family level. These results showed that the microbiota of *H. pylori*-positive children and adults showed a lower bacterial genus and family diversity than that of *H. pylori*-negative children and parents. In animal studies, *H. pylori* influenced the gastrointestinal microbiota and host immune responses not only locally in the stomach but also distantly as well, affecting important target organs (29). This is the first report that gastric infection with *H. pylori* affects the composition of intestinal microbiota in a human host.

Indigenous intestinal microbiota have an ability to protect host organisms against infection by exogenous pathogens (30). However, the role of endogenous intestinal microbiota in the transmission of *H. pylori* is as yet unknown. The origin of *H. pylori* in children is thought to originate from other family members, especially parents. If both the original infected family member and index child are found to have similar microbiota, it is possible that the protective effect by intestinal microbiota on the infection of *H. pylori* may be weak. Microbiota in the intestinal tract of a *H. pylori*-positive person may not have considerable amounts of bacteria that inhibit *H. pylori*. For example, bifidobacteria (31) and lactobacilli differ among individuals, and children inherit it from mother absolutely. Bifidobacteria and lactobacilli are dominant bacteria in infant (32) especially before weaning. It was also reported that *Lactobacillus* strains have an inhibitory effect against *H. pylori* (15, 33, 34).

In this study, we also focused on the similarity of the index child and other family members, especially *H. pylori*-positive members and *H. pylori*-negative siblings. In family 1, the microbiota of the index child was similar to that of the *H. pylori* negative mother; however, in the other four families, lowest Manhattan distances (most similar) were shown between index and *H. pylori*-infected mothers (three cases) and father (only one case). These data showed that the similarity of microbiota was related to transmission of *H. pylori* infection.

The Manhattan distances between *H. pylori*-negative siblings and *H. pylori*-positive parents were compared to those between index children and *H. pylori*-positive parents and shown to be shorter with the exception of family 2, where all three family members were similar to each other. In family 5, the *H. pylori*-positive index child was similar to the *H. pylori*-positive father, and the *H. pylori*-negative sibling showed a similar microbiome to that of the infected mother implying father-to-child transmission.

In conclusion, our data support the idea that the intestinal microbiota may contribute to intrafamilial transmission of *H. pylori* and that similarity of microbiota could be considered a risk factor. However, it is possible that the similarity in microbiota is also an effect o*f H. pylori* infection. This possibility is worthy of further exploration.

## Ethics Statement

This study was carried out in accordance with the recommendations of ethical guidelines for clinical research in Japan. All experiments were performed in accordance with the principles of Declaration of Helsinki with written informed consents. The study protocol (H22-047-02) was approved by the ethics committees of Kyorin University.

## Author Contributions

Conceived and designed the experiments: TO, SKikuchi, SKamiya, and MO. Wrote the paper: TO, CZ, YL, and SKamiya. Metagenome analysis: TO, EN, FH, and HY. Analyzed the data: TO and HY. Contributed reagents/materials/analysis tools: SKurata and TH.

## Conflict of Interest Statement

The authors declare that the research was conducted in the absence of any commercial or financial relationships that could be construed as a potential conflict of interest.
